# Optimization of combustion organization scheme for pre-combustion chamber of pre-cooled engine

**DOI:** 10.1038/s41598-023-43259-8

**Published:** 2023-09-25

**Authors:** Nanjia Yu, Bowei Jiao, Chuang Zhou, Shutao Han, Tianwen Li, Haoran Shi

**Affiliations:** School of Astronautics, Beihang University, Beijing, 100191 People’s Republic of China

**Keywords:** Aerospace engineering, Fluid dynamics

## Abstract

Pre-cooled engines, in which the incoming air is cooled by a pre-cooler before it enters the subsequent components for operation, are one of the important developments in combined power solutions. Therefore, how to optimize the gas temperature uniformity of the high temperature gas stream at the outlet of the pre-combustion chamber to achieve higher efficiency of the pre-cooled engine will be the main research content. In this paper, grid partitioning was performed on the pre combustion chamber model, and the k-omega model and EDC model were used to simulate the internal flow field of the pre combustion chamber. And verify the correctness of the simulation through engine hot testing. Explored the changing trends of the internal velocity and temperature fields of the engine under different secondary injection structures. The larger the secondary injection flow rate, the more obvious the obstruction to high-temperature gas, and the better the uniformity of gas temperature. However, in experiments, the secondary injection components often cannot withstand a large flow rate ratio. Ultimately, the gas temperature uniformity is best when the secondary injection flow rate ratio is 65%. Circumferential deflection will cause the gas to spin, and the spinning process will make the gas temperature at the same radius more uniform. However, due to the decrease in radial velocity, the obstruction effect on the overall high-temperature gas is weakened. When the gas is deflected towards the head by 30°, the velocity of the incoming gas and the velocity of the secondary injection gas are combined and perpendicular to the axis. At this time, the gas temperature uniformity is the best.

## Introduction

With the development of the times and the progress of society, the human demand for space exploration is increasing. Since entering the twenty-first century, space launch activities of space payloads around the world have become more and more active, and various new plans for exploring the universe are on the agenda. At present, space launch vehicles still mainly use one-time multi-stage launch vehicle systems^1^. Therefore, the world’s major spacefaring nations are actively developing a new generation of launch technologies, of which the pre-cooled engine is one of the important development directions in the space engine program. These engines cool the incoming air through a pre-cooler before it enters the subsequent components for work. Power units with pre-coolers can reduce the amount of oxidizer carried, significantly increase the engine specific impulse, improve the payload rate of the vehicle, and expand the engine’s operating envelope^2^.

According to the classification of the cold medium and air heat transfer, pre-cooling cycle engine can be divided into two categories of pre-cooling jet and heat exchanger pre-cooling. Among them, the pre-cooling cycle with heat exchanger can be divided into direct pre-cooling of fuel and indirect pre-cooling of intermediate mass according to whether the intermediate mass is used, as Table 1 shows the classification of pre-cooled engines.

**Table 1:** Pre-cooling engine classification.

MIPCC	Direct fuel pre-cooling	Indirect pre-cooling of intermediate working fluid
MIPCC	LACE	SABRE
Steam-jet	KLIN	SCIMITAR
	ATREX	

**Figure 1:**
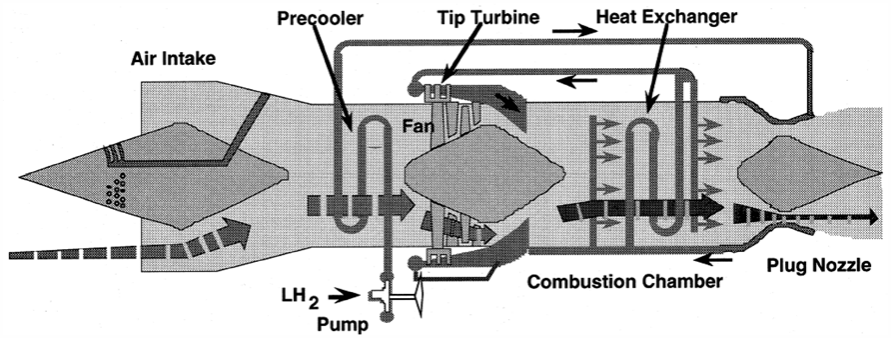
ATREX engine structure diagram.

The concept of mass injection and pre compressor cooling (MIPCC) first appeared in the 1950s. The basic idea of MIPCC^3^ is to inject water or oxidant into the incoming air before the compressor, and precool the incoming air to reduce its temperature. In 1986, research institutions such as the Institute of Space and Astronautical Science (ISAS) began developing ATREX engines for reusable Two Stage to Orbit (TSTO) space shuttles. ATREX is an expansion type air turbine ramjet engine that uses liquid hydrogen as fuel and coolant^4^. The structural diagram of the ATREX engine is shown in Fig. 1.

**Figure 2:**
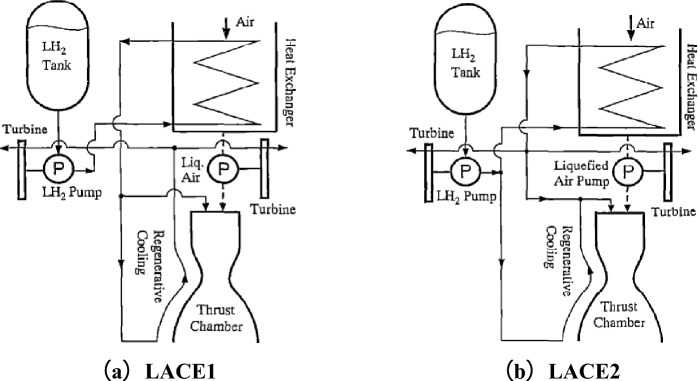
LACE basic model loop diagram.

The liquid air cycle engine (LACE), a pre cooled cycle engine based on heat exchangers, was first studied by Marquardt in the United States in the late 1950s. LACE has a high thrust to weight ratio and a wide Mach number operating range. Figure 2 shows the basic model cycle diagram of LACE, with main engine components including air hydrogen heat exchanger, liquid air pump, liquid hydrogen pump, turbine, and main thrust chamber. LACE uses liquid hydrogen to liquefy the air captured in the intake duct, and pumps the liquid air to the conventional rocket combustion chamber, where it reacts with hydrogen gas and is discharged.

In 1988, the British government withdrew funding from the space shuttle HOTOL project, and the research was interrupted. Alan Bond, who was involved in the research at the time^5^. The SKYLON project was powered by the SABRE, a modified version of the RB545 engine^6^. The powerplant for the SKYLON project was the SABRE, an improvement on the RB545 engine. Over the next three decades, the SKYLON project went through several iterations. The SABRE3 was the corresponding engine for the SKYLON C1 version^7^. The SABRE3 has both suction and rocket modes of operation, integrating a pre-cooled suction engine with a rocket engine, minimizing duplication of equipment^8–10^.

Gas generator^11^ the main function of the gas generator is to generate high temperature gas and high pressure gas, high temperature and high pressure gas has a very high energy, after further expansion can continue to do external work.

Gas generators can be divided into three categories based on the number of propellant elements^12^. Single-component gas generators, two-component gas generators and three-component gas generators. Single-component gas generators are mainly used in pilot systems, where the propellants are mainly hydrogen peroxide^13^ and nitrous oxide^14^ etc. The two-component gas generator is currently the most used in large liquid rocket engines, and its main function is to generate high-temperature gas. Fang et al.^15^ have successfully designed a pilot system based on an aero-engine by conducting experimental research on a new structure of gas generator with air/alcohol single nozzle. Dongying et al.^16^ conducted a thermal experimental study of a liquid oxygen/methane gas generator, and finally obtained that the gas generator design scheme and practical application are feasible and have good results. The three-component gas generator is more commonly used in ground test pilot systems, and its components are generally oxidizer, fuel and thermo regulator. Li^17^ in contrast, the spray combustion experiments of hydrogen/liquid oxygen/kerosene three-component gas generators are investigated by means of experiments and numerical simulations.

In this paper, we explore the best injection scheme to meet the working conditions by changing the angle of secondary injection and the percentage of secondary injection flow.

The shortcomings of this paper are: 1. affected by the size of the pre-combustion chamber structure, the angular deflection variation range is not large. 2. Affected by the flow rate, almost in the limit state before reaching a better gas temperature uniformity.

## Research model

Pre-combustion chamber uses gas-hydrogen-gas-oxygen torch type igniter to ignite, the combustion and mixing of gas–hydrogen–nitrogen–gas–oxygen three components, the pre-combustion chamber is divided into igniter, head, combustion section, mixing section, etc., where the outlet of mixing section is connected with the main combustion chamber.

**Figure 3:**
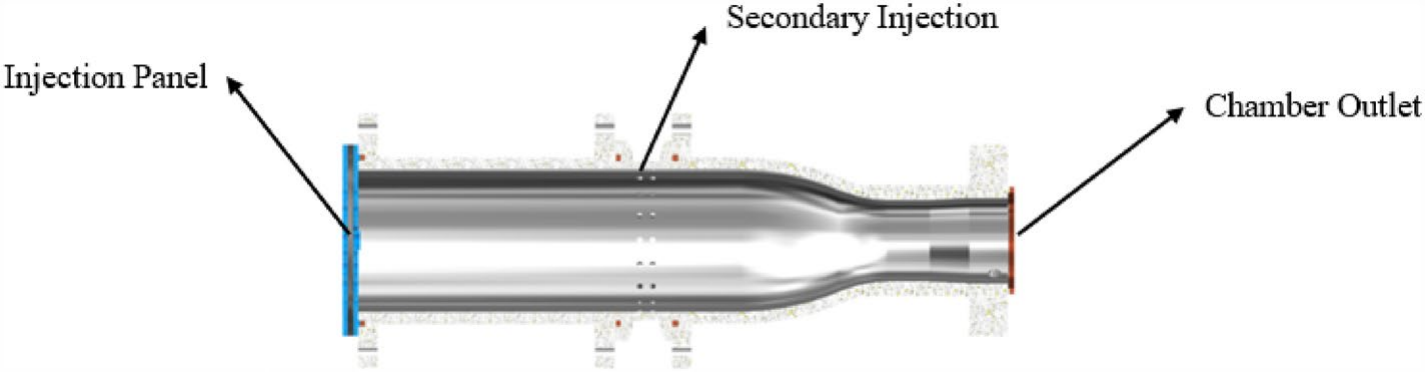
Half section view of pre-chamber.

**Figure 4:**
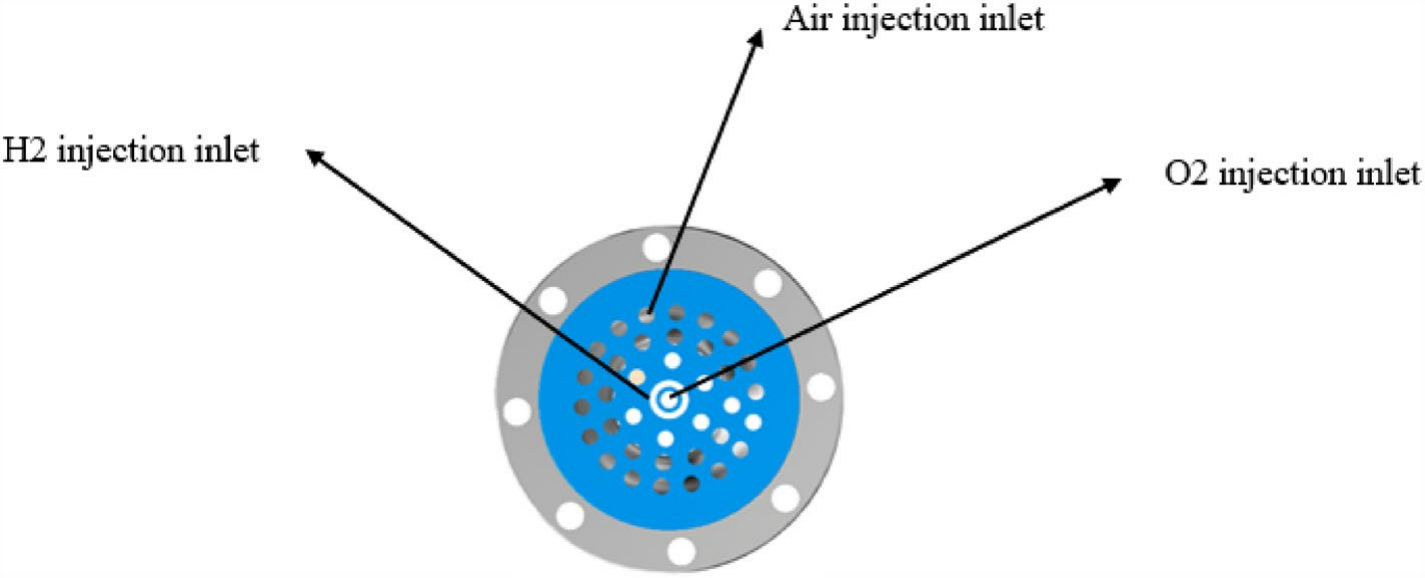
The injection panel to the pre-combustion chamber.

For the current pre-combustion chamber combustion is mostly more oxygen and less hydrogen, the overall plan of hydrogen zoning combustion is adopted: hydrogen is sprayed from the center of the pre-combustion chamber to form a high-temperature combustion zone with oxygen combustion, and air is sprayed from the surrounding combustion mixing. The surrounding air can protect the chamber wall of the pre-combustion chamber, which is not easy to contact with the high temperature gas, and is conducive to improving the structural safety. As Figs. 3 and 4 are the semi-sectional diagram of the pre-combustion chamber and the cross-sectional diagram of the pre-combustion chamber inlet.

### Brief description of secondary injection design

**Figure 5:**
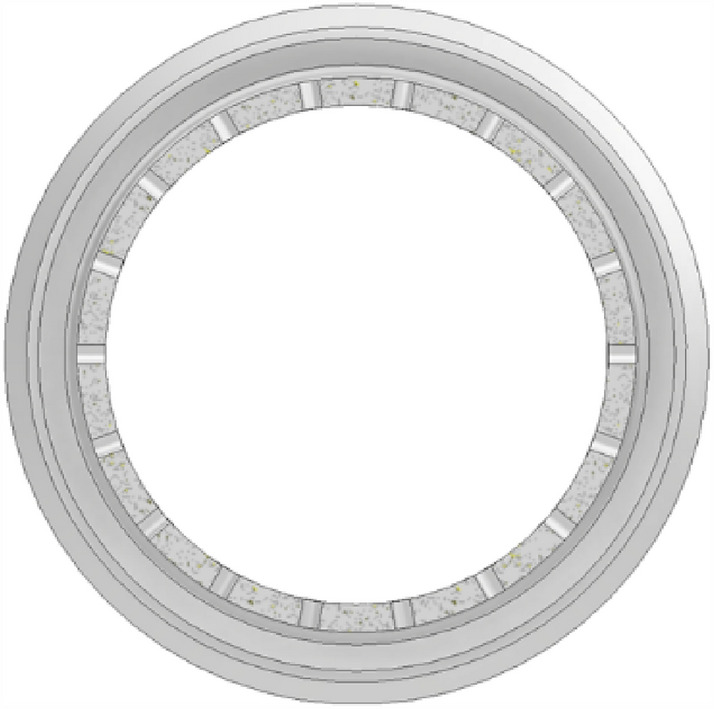
Radial half section view of secondary injection.

**Figure 6:**
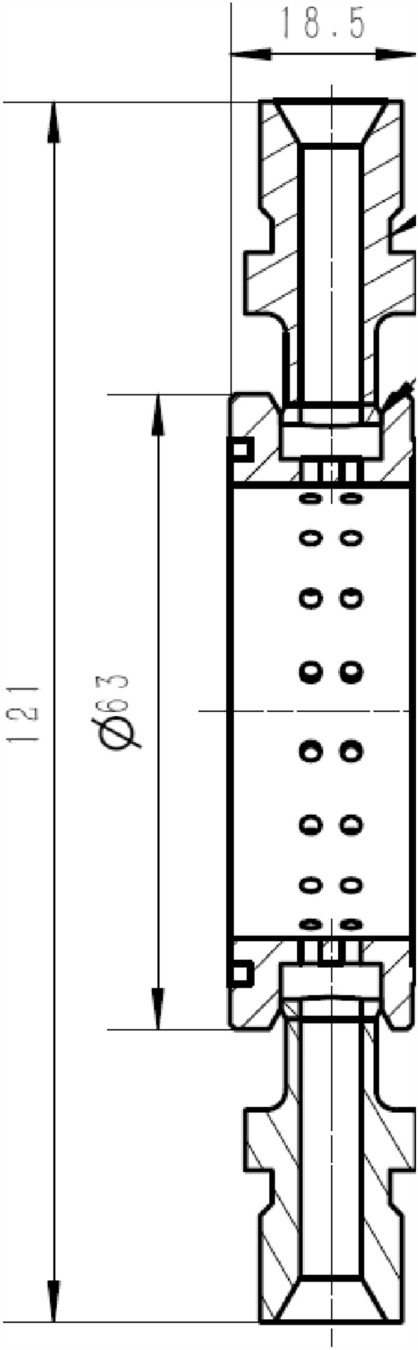
Body second injection diagram.

The combustion of hydrogen and oxygen in the center, the surrounding air combustion scheme, the hydrogen is mainly concentrated in the center of the pre-combustion chamber and helps to ensure a uniform distribution of gas. The middle of the pre-combustion chamber is used to inject nitrogen into the pre-combustion chamber through secondary injection. The main design parameters of the secondary injection are the number of injection holes in the body is generally 36, the body injection hole diameter is generally 1.9 mm, as Figs. 5 and 6 shows.

## Selection of numerical simulation models

### Gas phase control equation

A three-dimensional numerical simulation study was conducted on the combustion organization of the pre combustion chamber and main combustion chamber using commercial software Ansys Fluent 2021R1. The software solved the gas phase turbulent flow and combustion control equations through a three-dimensional grid. The N–S equation of the Reynolds mean conservation type for multi-component chemical reactions is established through the laws of mass conservation, momentum conservation, and energy conservation. The vector form of the three-dimensional unsteady compressible flow N–S control equation is as follows:

$$\frac{{\partial {\varvec{Q}}}}{\partial t} + \frac{{\partial \left( {{\varvec{E}} - {\varvec{E}}_{{\varvec{V}}} } \right)}}{\partial x} + \frac{{\partial \left( {{\varvec{F}} - {\varvec{F}}_{{\varvec{V}}} } \right)}}{\partial y} + \frac{{\partial \left( {{\varvec{G}} - {\varvec{G}}_{{\varvec{V}}} } \right)}}{\partial z} = {\varvec{H}}.$$

In the equation

$${\varvec{Q}} = \left[ {\begin{array}{*{20}c} {\begin{array}{*{20}c} \rho \\ {\rho u} \\ {\rho v} \\ {\rho w} \\ {\rho e} \\ \end{array} } \\ {\rho Y_{i} } \\ \end{array} } \right];\;{\varvec{E}} = \left[ {\begin{array}{*{20}c} {\begin{array}{*{20}c} {\rho u} \\ {\rho u^{2} + p} \\ {\rho uv} \\ {\rho uw} \\ {\left( {\rho e + p} \right)u} \\ \end{array} } \\ {\rho uY_{i} } \\ \end{array} } \right];\;{\varvec{F}} = \left[ {\begin{array}{*{20}c} {\begin{array}{*{20}c} {\rho v} \\ {\rho uv} \\ {\rho v^{2} + p} \\ {\rho vw} \\ {\left( {\rho e + p} \right)v} \\ \end{array} } \\ {\rho vY_{i} } \\ \end{array} } \right];\;{\varvec{G}} = \left[ {\begin{array}{*{20}c} {\begin{array}{*{20}c} {\rho w} \\ {\rho uw} \\ {\rho vw} \\ {\rho w^{2} + p} \\ {\left( {\rho e + p} \right)w} \\ \end{array} } \\ {\rho wY_{i} } \\ \end{array} } \right],$$

$${\varvec{H}} = \left[ {\begin{array}{*{20}c} 0 \\ 0 \\ 0 \\ 0 \\ 0 \\ {w_{i} } \\ \end{array} } \right];\;{\varvec{E}}_{{\varvec{V}}} = \left[ {\begin{array}{*{20}c} {\begin{array}{*{20}c} 0 \\ {\tau_{xx} } \\ {\tau_{xy} } \\ {\tau_{xz} } \\ {u\tau_{xx} + v\tau_{xy} + w\tau_{xz} - q_{x} } \\ \end{array} } \\ {\rho_{i} D_{im} \partial Y_{i} /\partial x} \\ \end{array} } \right],$$

$${\varvec{F}}_{{\varvec{V}}} = \left[ {\begin{array}{*{20}c} {\begin{array}{*{20}c} 0 \\ {\tau_{xy} } \\ {\tau_{yy} } \\ {\tau_{yz} } \\ {u\tau_{xy} + v\tau_{yy} + w\tau_{yz} - q_{y} } \\ \end{array} } \\ {\rho_{i} D_{im} \partial Y_{i} /\partial y} \\ \end{array} } \right],\;{\varvec{G}}_{{\varvec{V}}} = \left[ {\begin{array}{*{20}c} {\begin{array}{*{20}c} 0 \\ {\tau_{xz} } \\ {\tau_{yz} } \\ {\tau_{zz} } \\ {u\tau_{xz} + v\tau_{yz} + w\tau_{zz} - q_{z} } \\ \end{array} } \\ {\rho_{i} D_{im} \partial Y_{i} /\partial z} \\ \end{array} } \right].$$

Among them: represents the total number of components; is the density of the mixed gas; is the respective density values in the mixed gas, Corresponds to the velocity distribution on the coordinate axes,, respectively; Is the pressure; It is internal energy; Is viscous stress; It is the energy flux caused by heat conduction and component diffusion; The mass fraction of different components in the mixed gas; Is the mass generation rate corresponding to component i, It is the energy flux caused by heat conduction and component diffusion.

### Turbulence control equation

Model selection as ‘Realizable model’, the meaning of ‘Realizable’ is that the model satisfies certain mathematical limitations on Reynolds stress, which is consistent with the physical phenomena of turbulent flow. Compared with the Standard model, the Realizable model can meet specific mathematical limitations for Reynolds stress in turbulent flow, and is suitable for analyzing physical phenomena of rotational flow, free flow of injection and mixing layers, pipeline and boundary layer flow. The equations and equations are:

$$\frac{\partial }{\partial t}\left( {\rho k} \right) + \frac{\partial }{{\partial x_{j} }}\left( {\rho ku_{j} } \right) = \frac{\partial }{{\partial x_{j} }}\left[ {\left( {\mu + \frac{{\mu_{t} }}{{\sigma_{k} }}} \right)\frac{\partial k}{{\partial x_{j} }}} \right] + G_{k} + G_{b} - \rho \varepsilon - Y_{M} - S_{k} ,$$

$$\begin{aligned} \frac{\partial }{\partial t}\left( {\rho \varepsilon } \right) + \frac{\partial }{{\partial x_{j} }}\left( {\rho \varepsilon u_{j} } \right) = & \frac{\partial }{{\partial x_{j} }}\left[ {\left( {\mu + \frac{{\mu_{t} }}{{\sigma_{k} }}} \right)\frac{\partial \varepsilon }{{\partial x_{j} }}} \right] \\ & + \rho C_{1} S\varepsilon - \rho C_{2} \frac{{\varepsilon^{2} }}{{k + \sqrt {v\varepsilon } }} + C_{1\varepsilon } \frac{\varepsilon }{k}C_{3\varepsilon } G_{b} + S_{\varepsilon } . \\ \end{aligned}$$

Calculate $\mu_{t}$ and $C_{\mu }$ in the formula as follows

$$\mu_{t} = \rho C_{\mu } \frac{{k^{2} }}{\varepsilon },$$

$$C_{\mu } = \frac{1}{{A_{0} + A_{s} U^{*} k/\varepsilon k}}.$$

In the equation: $$C_{1} = \max \left[ {0.43,\frac{\eta }{\eta + 5}} \right]$$, $$\eta = S\frac{k}{\varepsilon }$$, $$S = \sqrt {2S_{ij} S_{ij} }$$, $$C_{1\varepsilon } = 1.44$$, $$C_{2} = 1.9$$$$\sigma_{k} = 1.0$$, $$\sigma_{\varepsilon } = 1.2.$$

### Chemical kinetic model

The combustion model is selected as the EDC model.The Eddy Dissipation Conceptual Model assumes that chemical reactions occur within the fine structure of each grid unit, which continuously exchanges energy and mass with the surrounding turbulent structure. Therefore, the RANS average variable of this grid unit can be expressed by the spatial scale of the fine structure and its relationship with the scalar transfer rate of the surrounding flow structure. The EDC model can incorporate detailed chemical reaction mechanisms into the turbulent reaction flow, but this can also cause rigidity issues in the equation, and the cost of numerical integration is high. Therefore, this model is usually used when the assumption of rapid chemical reactions is invalid.

## Experimental validation

**Figure 7:**
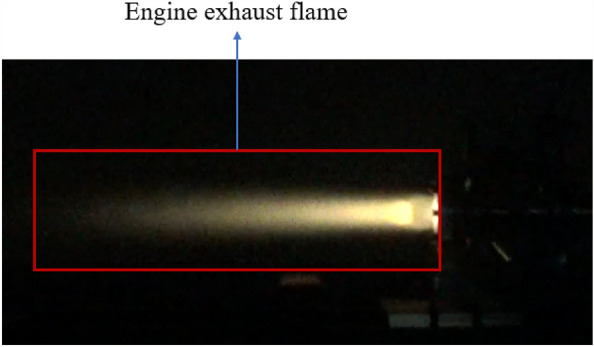
Pre-combustion chamber hot commissioning.

**Figure 8:**
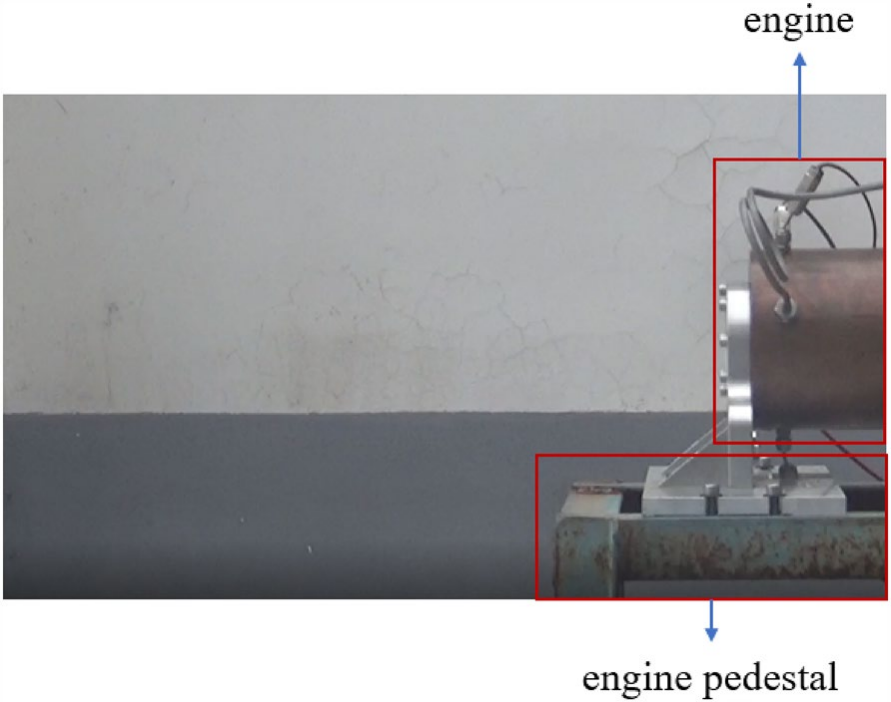
Engine testing site.

**Figure 9:**
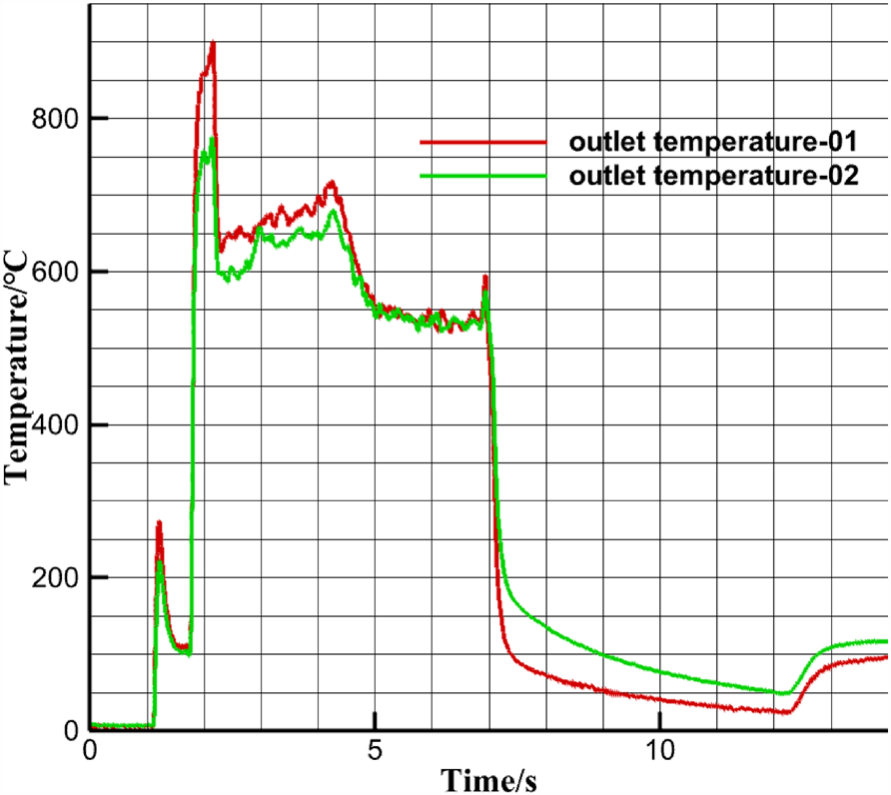
Pre-combustion chamber outlet temperature.

Nanjia’s group at the School of Astronautics, Beihang University conducted a hot commissioning of the pre-combustion chamber, and selected the combustion temperature near the outlet wall of the pre-combustion chamber as the basis for the simulation verification, and the experimental site is shown in Figs. 7, 8 The outlet temperature curve is shown in Fig. 9, and the working time sequence is shown in Table 3. The experimental working conditions are shown in Table 2.

**Table 2:** Hot commissioning conditions table.

H2/g/s	O2/g/s	Air/g/s	N2/g/s	Chamber pressure/MPa	Outlet pressure/MPa	Proportion of secondary injection flow (%)
6.3	101.0	468.6	246.8	1.2	0.35	30

According to Fig. 9 and Table 3 The analysis shows that the pre-combustion chamber for oxygen-rich ignition to prevent deflagration, the spark plug ignition at 1.1 s, the pre-combustion chamber outlet near wall combustion temperature has a small increase in 1.6 s when the head of the pre-combustion chamber oxygen solenoid valve open, the pre-combustion chamber outlet near wall combustion temperature rose to 900 K, pre-combustion chamber combustion, at 2.1 s, the secondary injection solenoid valve open, the temperature back down, at 4.1 s when the igniter spark plug off, pre-combustion chamber Work alone for 2.5 s, the pre-combustion chamber near the wall surface combustion temperature reaches a flat section of 550 K, and record this temperature for the pre-combustion chamber outlet working temperature.

**Table 3:** Pre-combustion chamber hot commissioning sequence.

Timing steps	Absolute time (ms)	Relative time interval (ms)	Control objects	Control volume
1	0	0	PXI trigger	Open
2	1000	1000	Igniter oxygen circuit before spraying solenoid valve—O2	Open
3	1100	100	Spark plug ignition 01	Open
4	1100	0	Igniter hydrogen circuit before the spray solenoid valve-H2	Open
5	1100	0	Nitrogen circuit 1 mixed before pneumatic	Open
6	1600	500	Main oxygen circuit 1 mixing front pneumatic	Open
7	1600	0	Main oxygen circuit 2 pneumatic before spraying	Open
8	2100	500	Nitrogen circuit 2 spray before pneumatic	Open
9	4100	2000	Igniter oxygen road before the spray solenoid valve-02	Off
10	4100	0	Spark plug ignition 01	Off
11	6600	2500	Main oxygen circuit 2 pneumatic before spraying	Off
12	6600	0	Main oxygen path 2 blow off before spraying	Open
13	6600	0	Main oxygen circuit 1 mixing front pneumatic	Off
14	7100	500	Igniter hydrogen circuit before the spray solenoid valve-H2	Off
15	7100	0	Igniter Oxygen pre-injection blow-off-O2	Open
16	7100	0	Blow-off-H2 before point hydrogen injection	Open
17	12,100	5000	Blow-off-H2 before point hydrogen injection	Off
18	12,100	0	Igniter Oxygen pre-injection blow-off-O2	Off
19	12,100	0	Nitrogen circuit 2 spray before pneumatic	Off
20	12,100	0	Nitrogen circuit 1 mixing front pneumatic	Off
21	12,100	0	Main oxygen path 2 blow off before spraying	Off
22	13,100	1000	PXI trigger	Off

**Figure 10:**
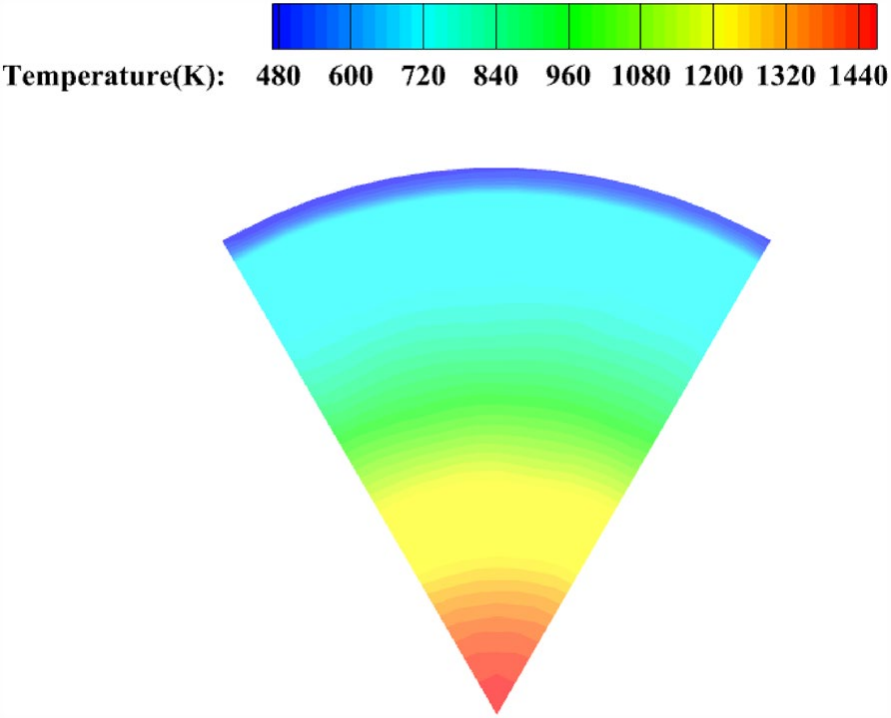
30 ratio outlet temperature graph.

The simulation results under this condition are as follows Fig. 10 The average temperature near the outlet wall is 530 K, which is approximately the same as the experimental result of 550 K. The error is within 5%, which can verify the authenticity of the simulation through the experiment.

## Results and discussion

### Simulation and analysis of for the percentage of secondary injection flow

#### Working conditions

As shown in Table 4, it is a simulation working condition table.

**Table 4:** Simulation conditions.

Number	H2/g/s	O2/g/s	Air/g/s	N2/g/s	Chamber pressure	Outlet pressure	Proportion of secondary injection flow (%)
1	6.3	101.0	468.6	246.8	1.2	0.35	30
2	6.3	110.3	428.5	329.1	1.2	0.35	40
3	6.3	150.7	254.3	411.4	1.2	0.35	50
4	6.3	175.6	147.2	493.6	1.2	0.35	60
5	6.3	188.0	93.6	534.8	1.2	0.35	65
6	6.3	209.7	0	606.7	1.2	0.35	73.7

#### Thermal calculation results

According to the CEA calculation program, the theoretical outlet temperature of the pre-combustion chamber is calculated as follows is shown in Table 5.

**Table 5:** Thermal calculation results.

H2/g/s	O2/g/s	N2/g/s	Outlet temperature (K)
6.3	209.7	606.7	855.1

#### Simulation results

##### Pre-combustion chamber cross-sectional temperature cloud

The simulation results for different secondary injection flow ratios in the pre-combustion chamber are shown in the following figures.

**Figure 11:**
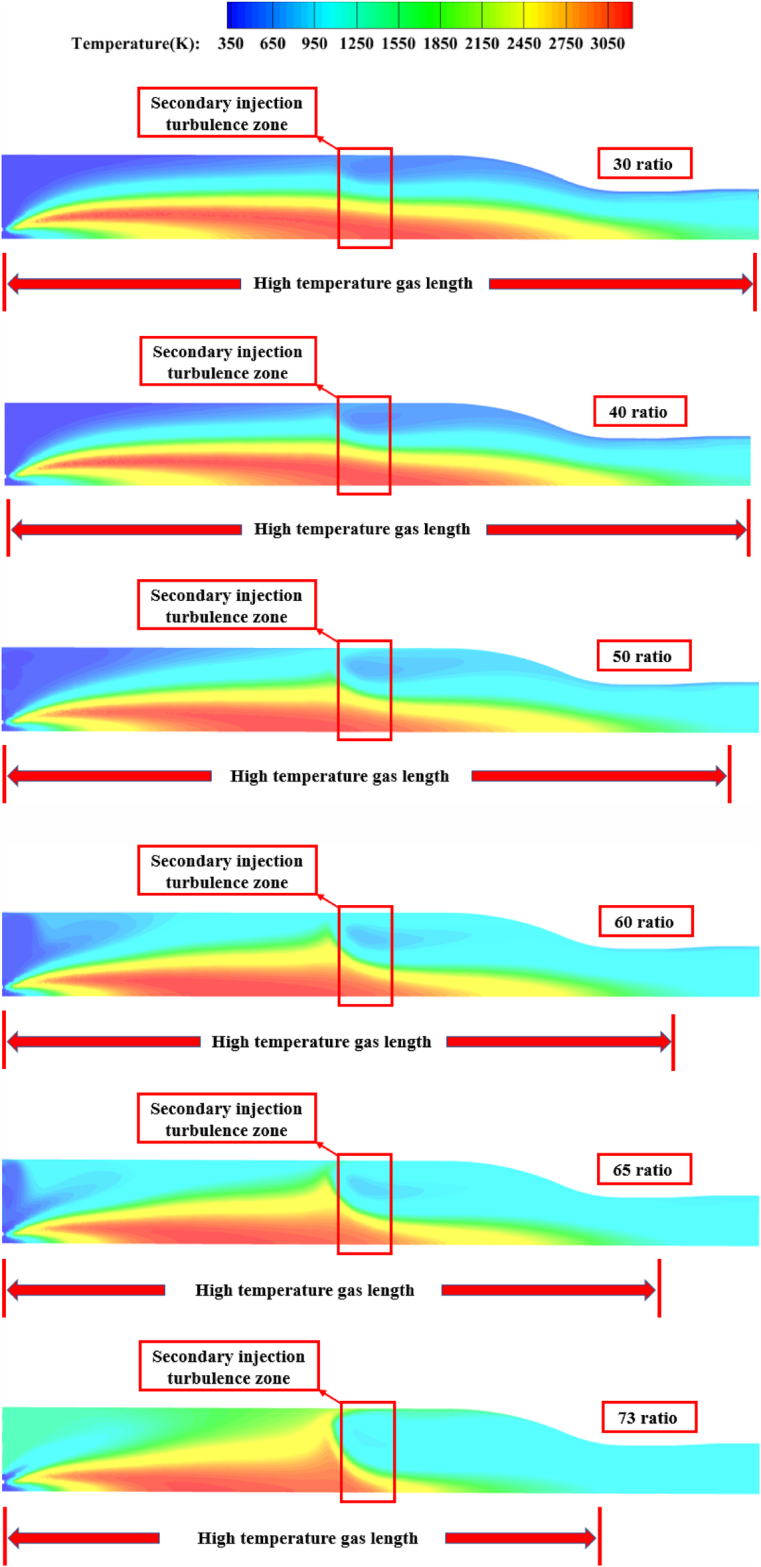
Pre-combustion chamber internal flow field temperature cloud diagram.

As shown in Fig. 11, as the percentage of the secondary injection flow increases, the length of the high temperature gas is shorter and the effect of the secondary flow on the obstruction of the head high temperature gas is more obvious, when the flow percentage is 30% and 40%, part of the high temperature gas stream can still reach the outlet of the pre-combustion chamber from the central area, and when the flow percentage is higher, the influence of the high temperature gas stream on the outlet temperature of the pre-combustion chamber decreases, and when the limit flow percentage is reached, the high temperature gas stream has almost no influence on the outlet temperature uniformity The temperature uniformity is considered to be the best at this time.

##### Pre-combustion chamber outlet temperature cross-sectional cloud diagram

**Figure 12:**
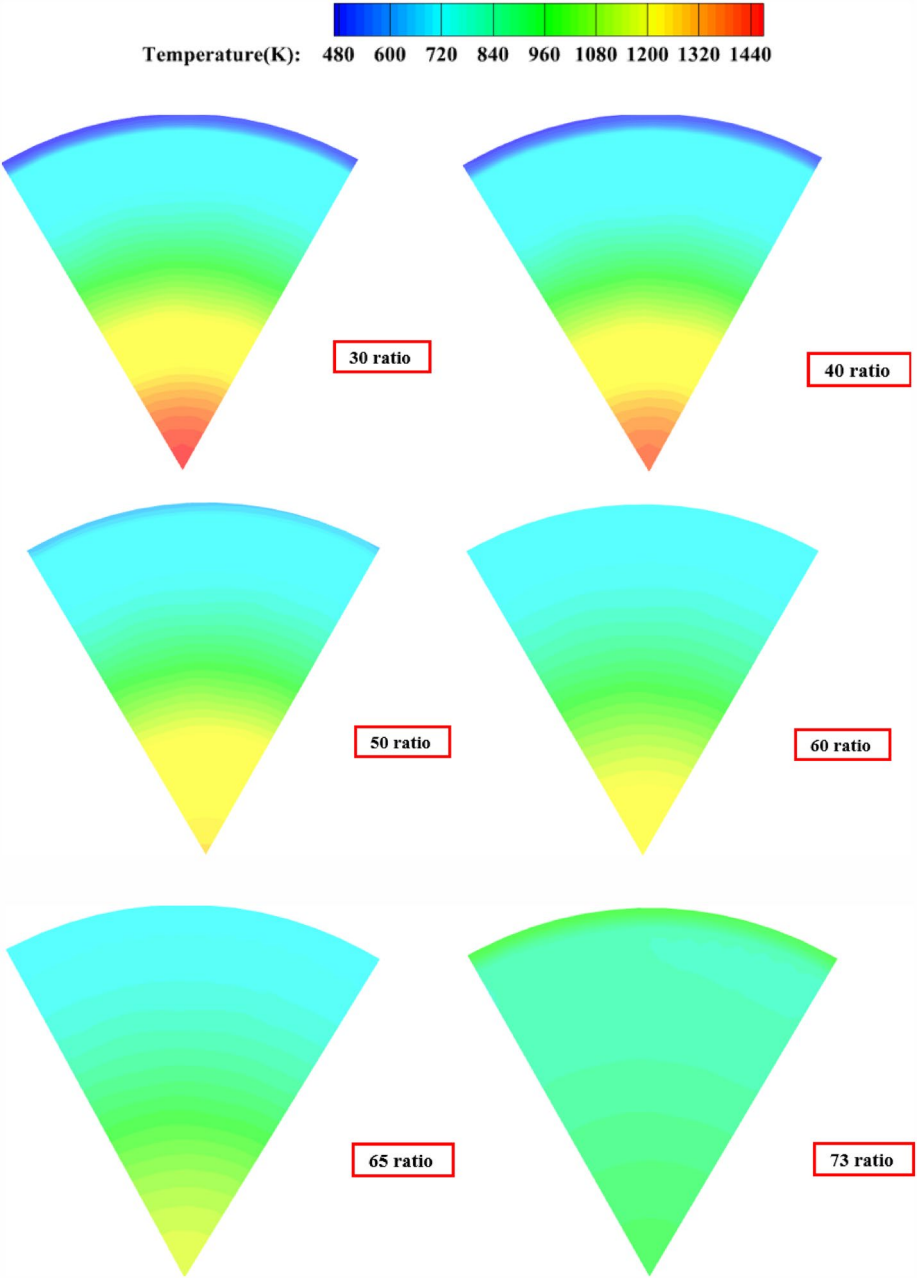
Pre-combustion chamber outlet temperature cloud.

As shown in Fig. 12, when the percentage of secondary injection flow is 30% and 40%, the high temperature airflow (red part) appears at the outlet of the pre-combustion chamber, and the temperature uniformity at the outlet of the pre-combustion chamber is poor, and the temperature change at the outlet of the pre-combustion chamber gradually decreases as the percentage of secondary injection flow increases, and the temperature uniformity at the outlet is best when the percentage of secondary injection flow reaches the limit.

##### Pre-combustion chamber internal flow field distribution cloud diagram

**Figure 13:**
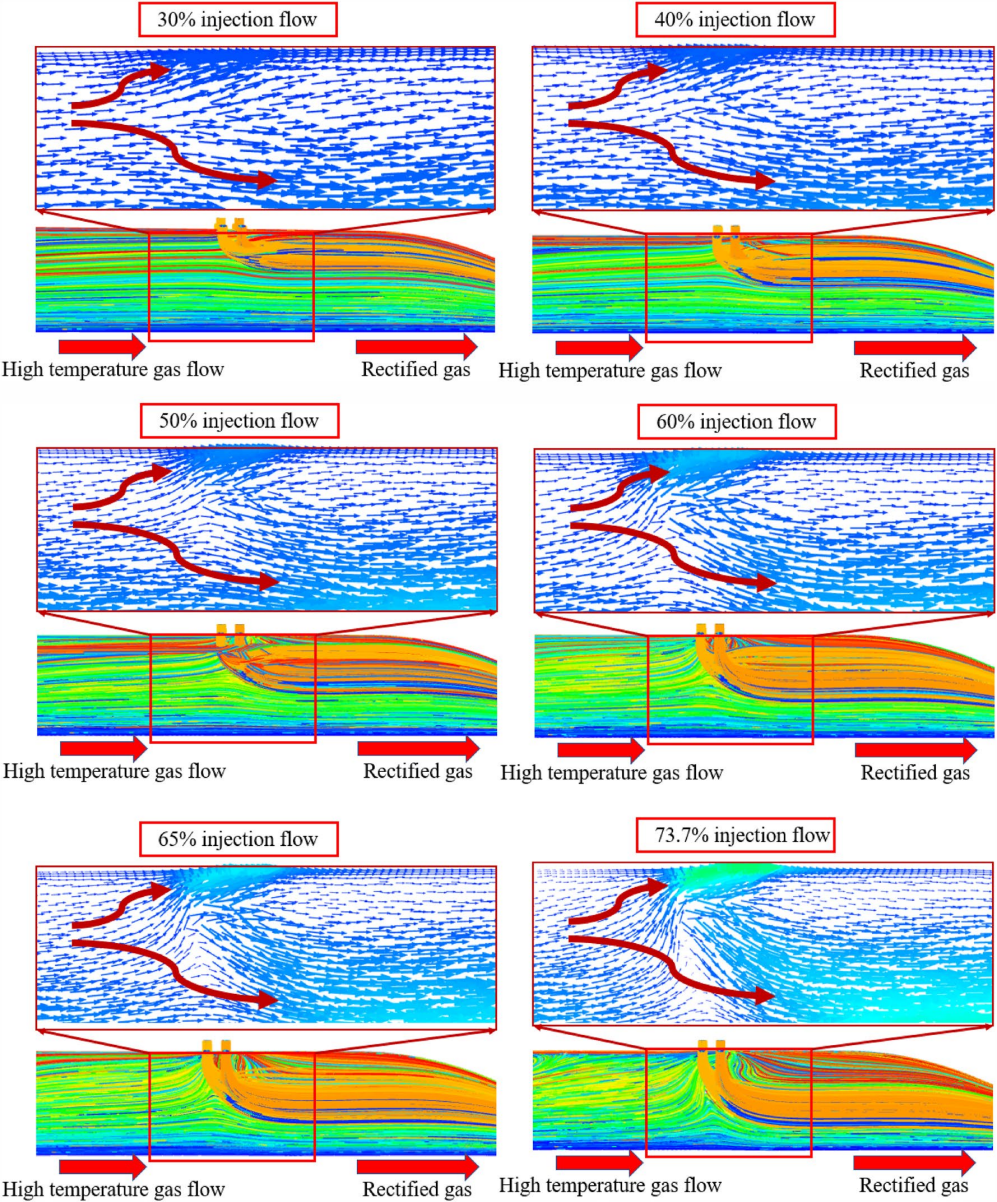
Pre-combustion internal flow field distribution trace diagram.

As shown in Fig. 13, the propellant ignites at the head of the engine, and after gas expansion, the flow direction gradually becomes horizontal. When approaching the position of the secondary injection, due to the higher flow rate of the secondary injection gas, according to Bernoulli’s principle, the greater the flow rate, the smaller the pressure, leading to an upward movement trend of the gas. As the proportion of the secondary injection flow rate increases, the momentum ratio of the secondary injection gas to the gas increases, Forcing high-temperature gas to move towards the center of the engine and reducing its axial distance, the secondary injection gas that does not participate in combustion also has a cooling effect on high-temperature gas. Under the combined effect, it will improve the uniformity of the engine outlet temperature.

Select 20 temperature distribution points at equal distance on a radius of the pre-combustion chamber outlet section and draw the following temperature distribution curve.

**Figure 14:**
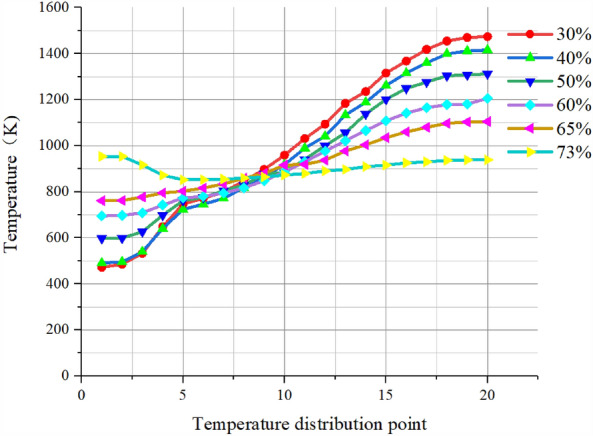
Temperature curve of secondary injection flow rate.

As shown in Fig. 14, with the increase of the percentage of secondary injection flow, the temperature uniformity is better, the curve is smoother, and the difference between the highest and lowest temperature on the radius of the outlet section of the pre-combustion chamber is reduced, which proves that increasing the percentage of secondary injection flow has an optimal effect on the temperature uniformity.

#### Simulation results analysis and conclusion

The OTDF (outlet temperature distribution factor) and standard deviation are calculated by selecting the simulation data points of the pre-combustion chamber outlet section and using Eqs. (1) and (2), respectively, as the criteria for outlet gas temperature uniformity.

1 $$\tau_{m} = \frac{{t_{\max } - \overline{t} }}{{\overline{t} }},$$

where ${\uptau }_{\mathrm{m}}$ is the OTDF value, and ${\mathrm{t}}_{\mathrm{max}}$ is the peak gas temperature, and $\overline{\mathrm{t} }$ is the average gas temperature.

2 $$S = \sqrt {\frac{{\sum\nolimits_{i = 1}^{n} {\left( {S_{i} - \overline{S} } \right)} }}{n}} ,$$

where $\mathrm{S}$ is the standard deviation, and $\mathrm{si}$ is the temperature at each point, and $\overline{\mathrm{S} }$ is the mean value of temperature, and n is the number of temperature samples.

**Figure 15:**
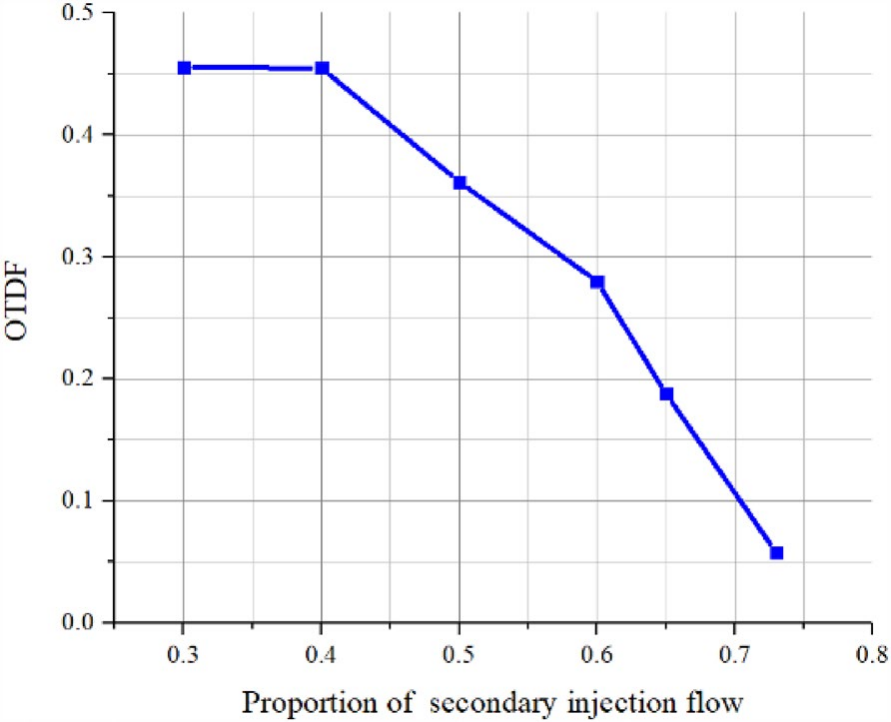
OTDF change curve with the proportion of secondary flow.

**Figure 16:**
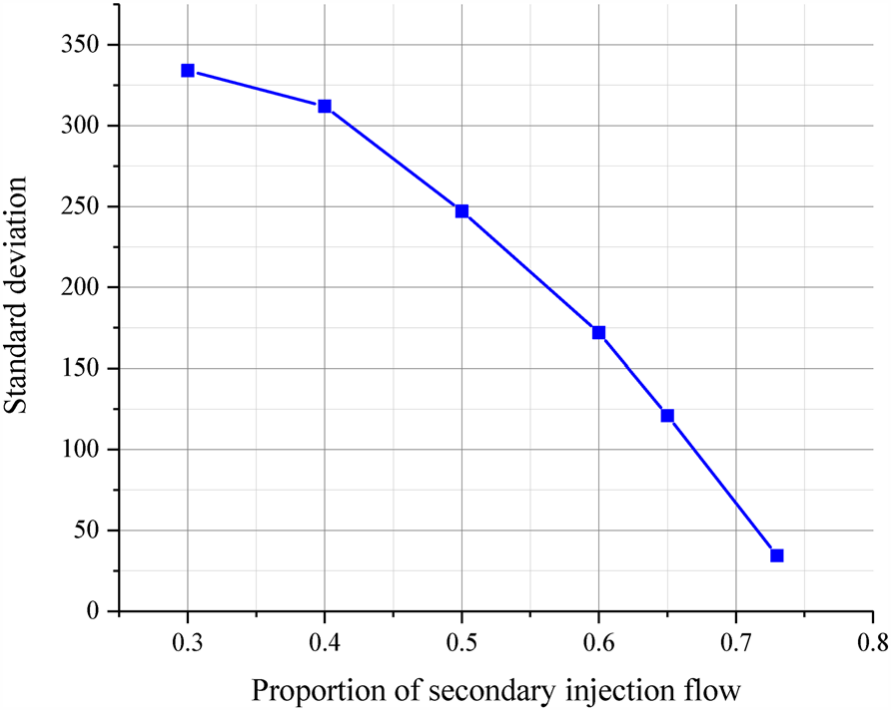
Standard deviation change curve with the proportion of secondary flow.

Table 6 shows the calculation results of simulation data.According to Table 6, plot the relationship between OTDF and standard deviation with the proportion of secondary injection flow rate, as shown in Figs. 15 and 16.

**Table 6:** Simulation result data.

Number	Proportion of secondary injection flow (%)	Temperature (K)	OTDF	Standard deviation
1	30	1010	0.4554	334
2	40	976	0.4549	312
3	50	962	0.3617	247
4	60	922	0.2798	172
5	65	926	0.1879	121
6	73	901	0.0577	34.4

### Simulation study and analysis of injection angle for secondary injection

#### Secondary injection for circumferential deflection

In order to investigate the effect of cyclonic flow on the uniformity of outlet gas temperature, the secondary injection was deflected circumferentially and varied at different angles, and the case of 65% of the secondary injection was selected for simulation study.

**Figure 17:**
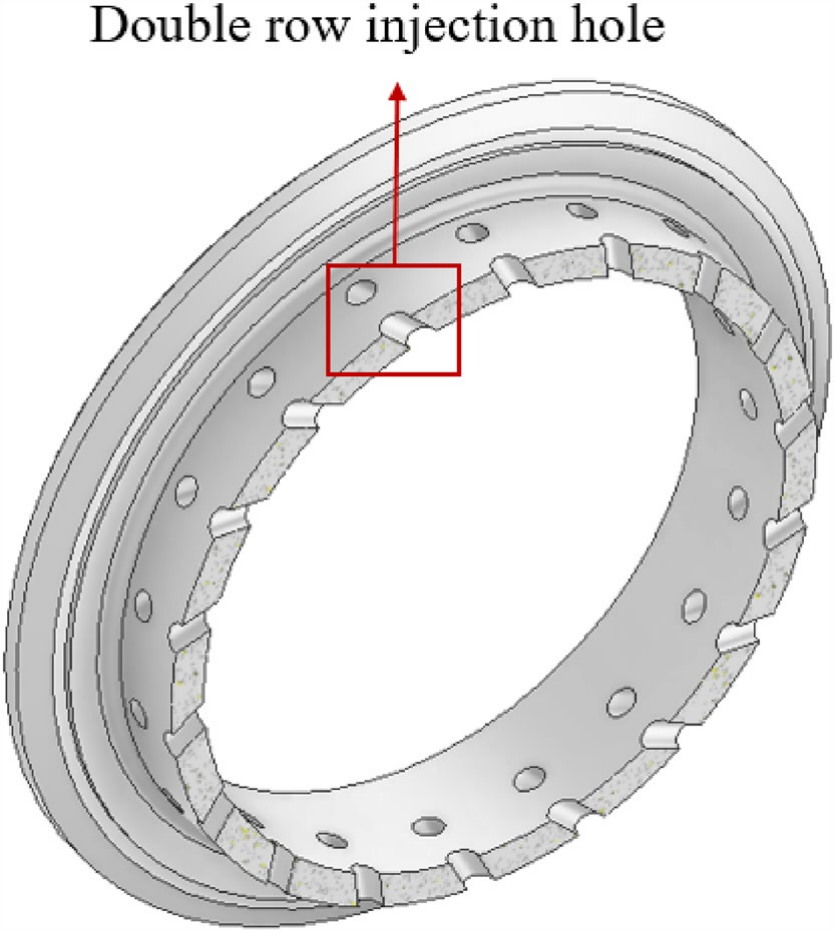
Secondary injection components (circumferential deflection)-1.

**Figure 18:**
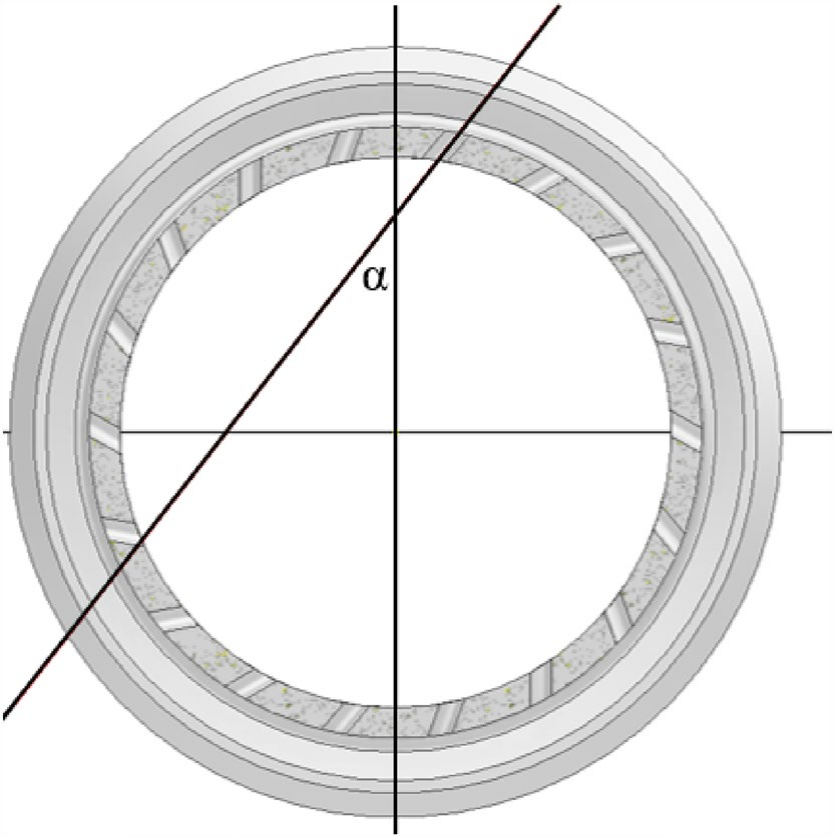
Secondary injection components (circumferential deflection)-2.

As shown in Figs. 17 and 18, the central injection structure (circumferential deflection). The central injection component (circumferential deflection) has two rows and 36 injection holes, as shown in Fig. 17. The angle α represents the angle between the injection hole and the radial direction, and four injection components with α values of 0°, 15°, 30°, and 45° were designed. According to Table 7, it is the simulation condition of the simulation for the secondary injection for circumferential deflection.

**Table 7:** Simulation condition.

Number	Proportion of secondary injection flow (%)	Injection angle (°)
1	65	0
2	65	15
3	65	30
4	65	45

The following are the simulation results and analysis: For example, the simulation results to explore the uniformity of the gas temperature at the outlet of the pre-combustion chamber by the gas start spin.

##### Pre-combustion chamber cross-sectional temperature cloud

**Figure 19:**
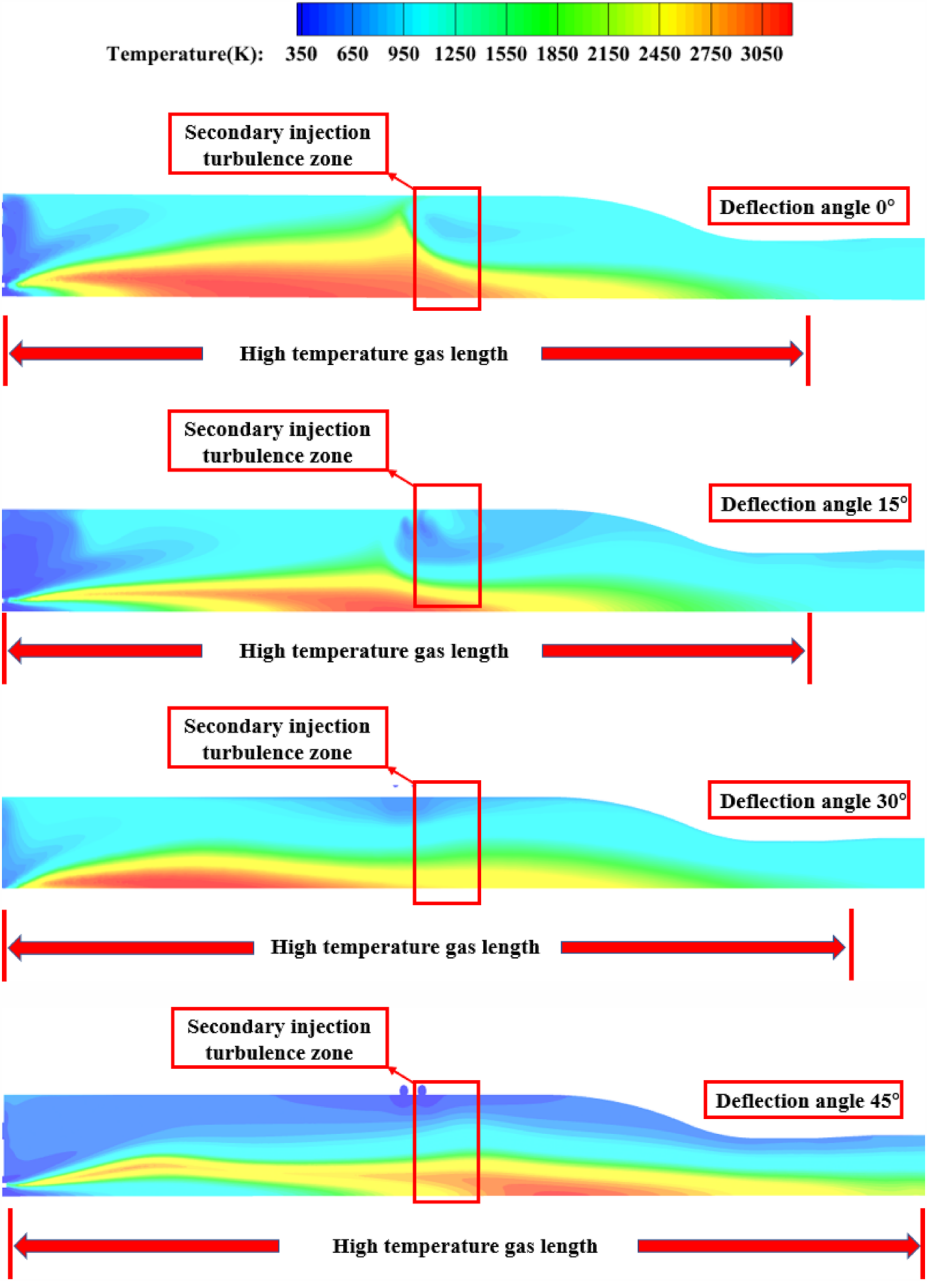
Pre-combustion chamber internal flow field temperature cloud diagram.

As Fig. 19 shown, the secondary injection injection angle after circumferential deflection, will weaken the secondary injection of high temperature incoming flow obstruction effect, with the angle increases, a large number of high-temperature gas flow through the secondary injection section will affect the temperature uniformity of the pre-combustion chamber outlet gas.

##### Pre-combustion chamber outlet temperature cross-sectional cloud diagram

**Figure 20:**
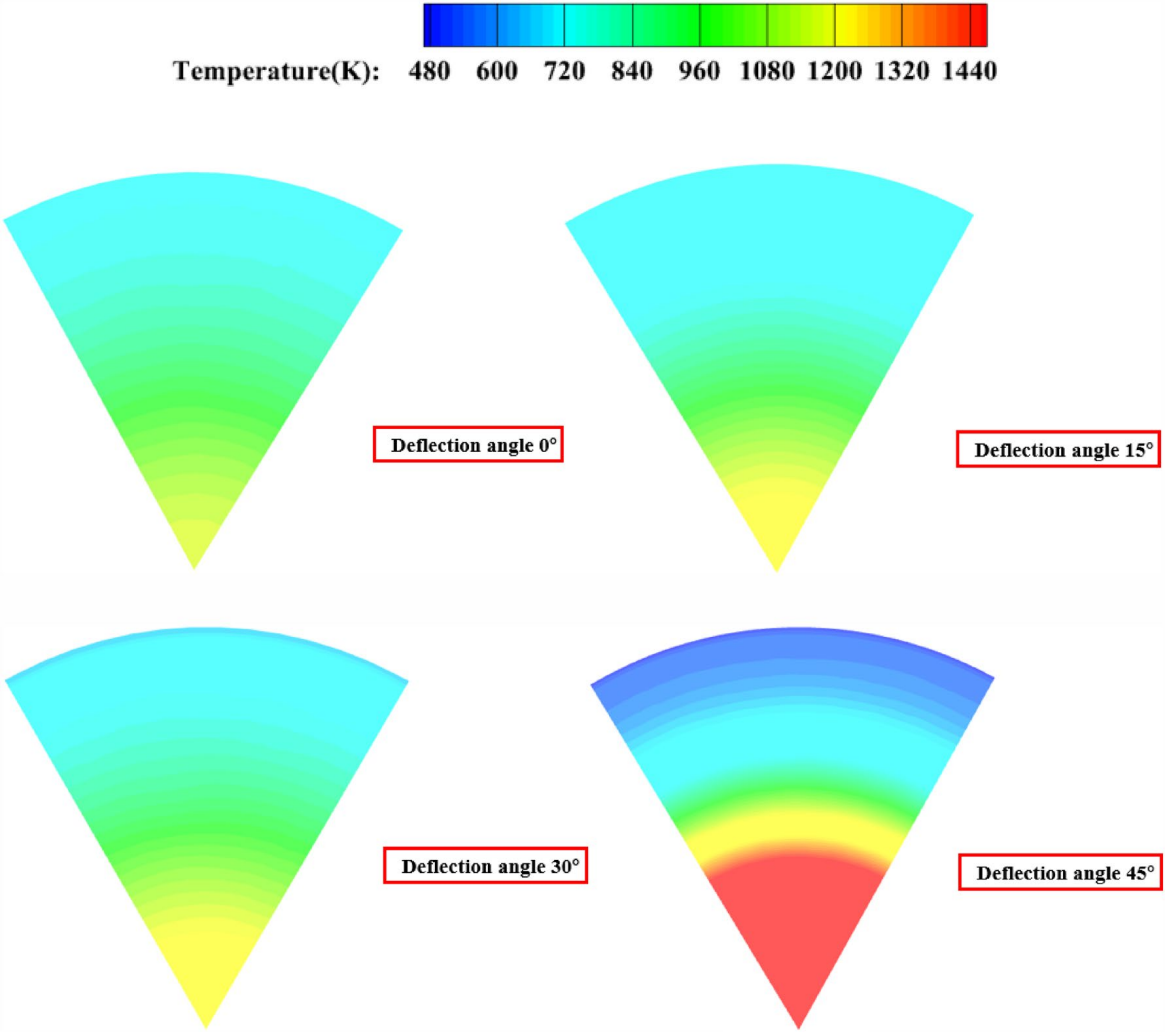
The static temperature cloud diagram of the outlet of the pre-combustion chamber with the injection angle of 30° 60° for the secondary injection; (**a**) injection angle of secondary injection 0°; (**b**) Injection angle of secondary injection 15°; (**c**) injection angle of secondary injection 30°; (**d**) injection angle of secondary injection 45°.

As shown in Fig. 20, when the secondary injection injection angle is 15°, 30° and 45°, the temperature uniformity of the gas ing the pre-combustion chamber is not as good as when the secondary injection is injected in the vertical axis direction.

##### Pre-combustion chamber internal flow field distribution cloud diagram

**Figure 21:**
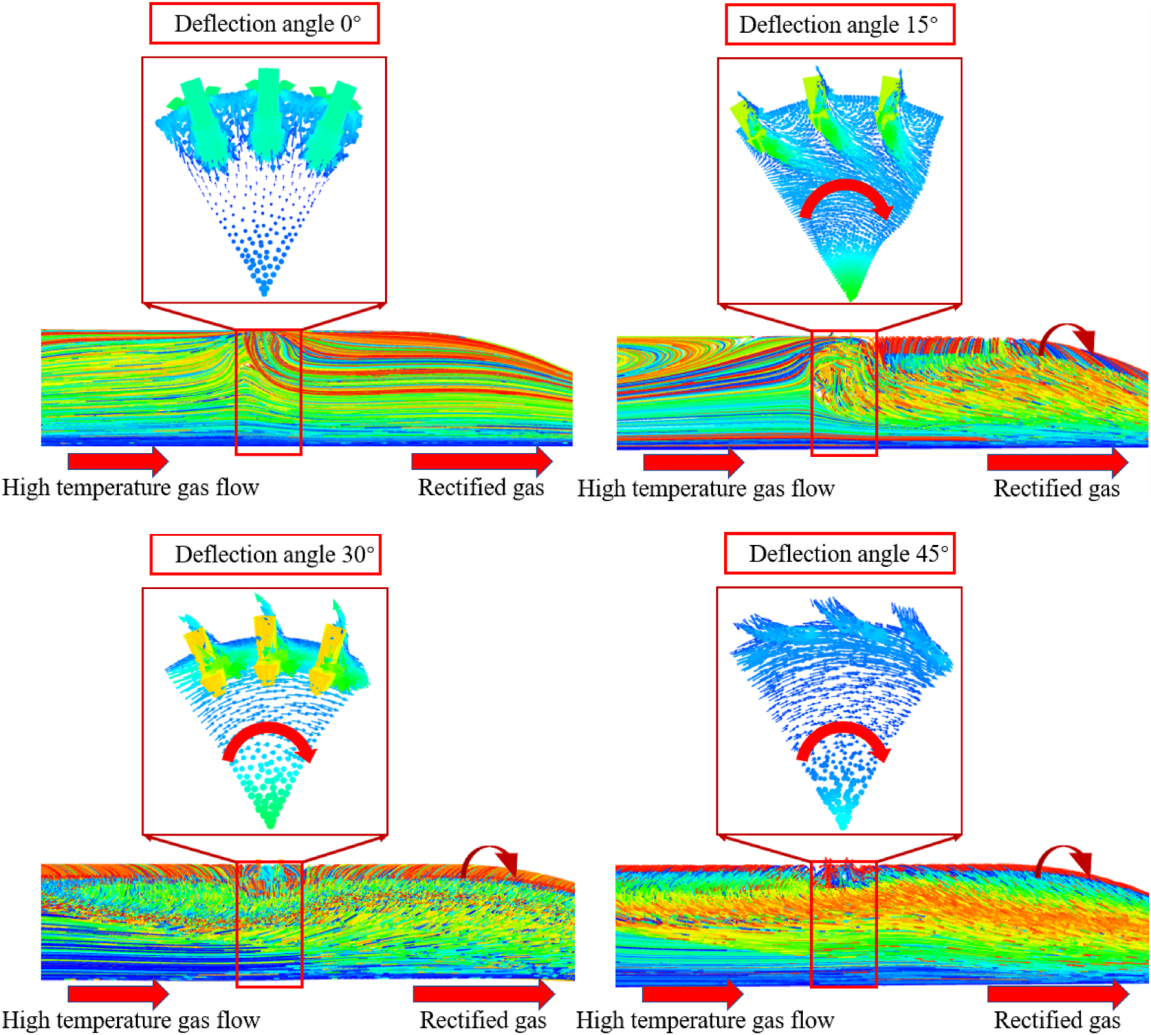
Pre-combustion chamber internal flow field distribution cloud diagram.

As shown in Fig. 21, the cloud diagram shows the changes in the internal flow field of the engine under different secondary injection angles. When the secondary injection angle increases from 0° to 45°, it is found that the swirl of high-temperature gas continues to increase. When the secondary injection angle is 15°, due to the swirling state of the secondary injection gas, it is mixed with the horizontal incoming gas to generate a vortex field below the secondary injection. After gradual rectification, a regular swirling gas is generated at the rear of the engine. Due to the small deflection angle, the secondary injection gas has a small swirling degree, and the radial velocity of the secondary injection gas is not significantly different from the undeflected state, resulting in little difference in outlet gas temperature uniformity. When the deflection angles are 30° and 45°, the vorticity is greater. High temperature gas and secondary injection gas not only generate intense vortex zones at the secondary injection, but also gradually affect the entire engine after rectification. The vortex gas is discharged from the head to the outlet near the upper wall of the engine. Due to the increase in injection angle, the radial velocity of the secondary injection gas is smaller, resulting in some high-temperature gas being directly ejected from the outlet from the center of the engine, resulting in a decrease in the uniformity of the outlet gas temperature.

By selecting the temperature distribution points on one radius of the pre-combustion chamber outlet section, the following temperature distribution profile was plotted.

**Figure 22:**
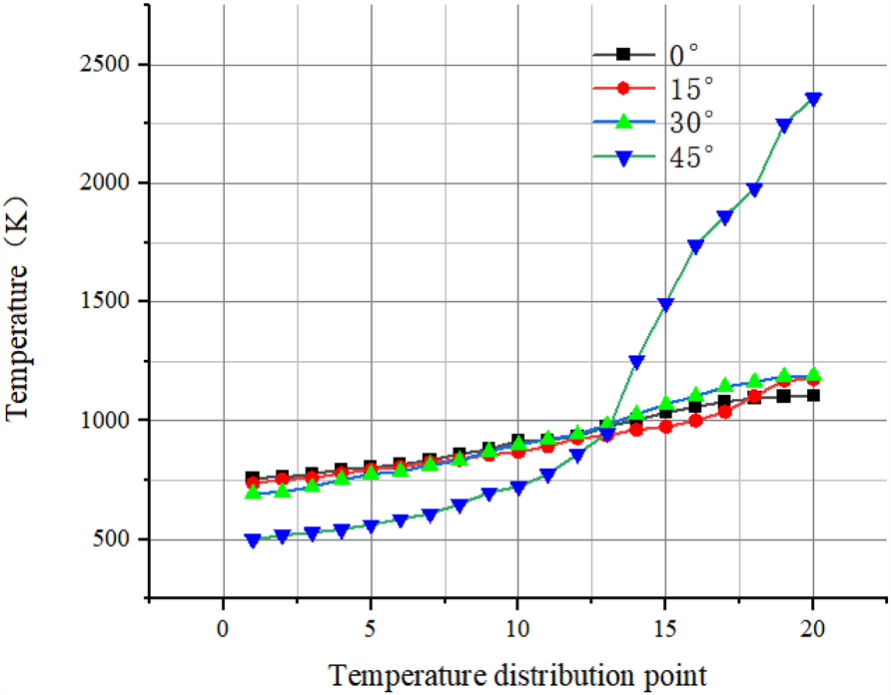
Temperature graph.

As Fig. 22 shown, the secondary injection with circumferential deflection does not have an optimized effect on the outlet gas temperature uniformity.

The following table shows the results of simulation calculations.

As Table 8 As shown, when the secondary injection injection angle for the circumferential deflection 0°, 15° and 30° injection, the average static temperature distribution in the vicinity of 920 K, when the deflection angle of 45°, the average static temperature increased to 1070 K, for the outlet gas temperature distribution coefficient deflection angle 0° when the coefficient is the lowest 0.1879, when the deflection angle of 15° and 30° when the coefficient is about 0.28, when the deflection angle of 45° when the coefficient increases to 1.206, the standard deviation increases with the angle from 121 to 634, indicating that when the larger the circumferential deflection angle, the worse the effect of secondary injection, when the secondary injection perpendicular to the axial injection, the outlet gas temperature uniformity is the best, and when the secondary injection angle reaches 45°, it can be considered that the secondary injection failure.

**Table 8:** Simulation result data.

Number	Injection angle (°)	Temperature (K)	OTDF	Standard deviation
1	0	926	0.1879	121
2	15	910	0.2967	135
3	30	929	0.2809	170
4	45	1070	1.206	634

#### Deflection angle for axial deflection

**Figure 23:**
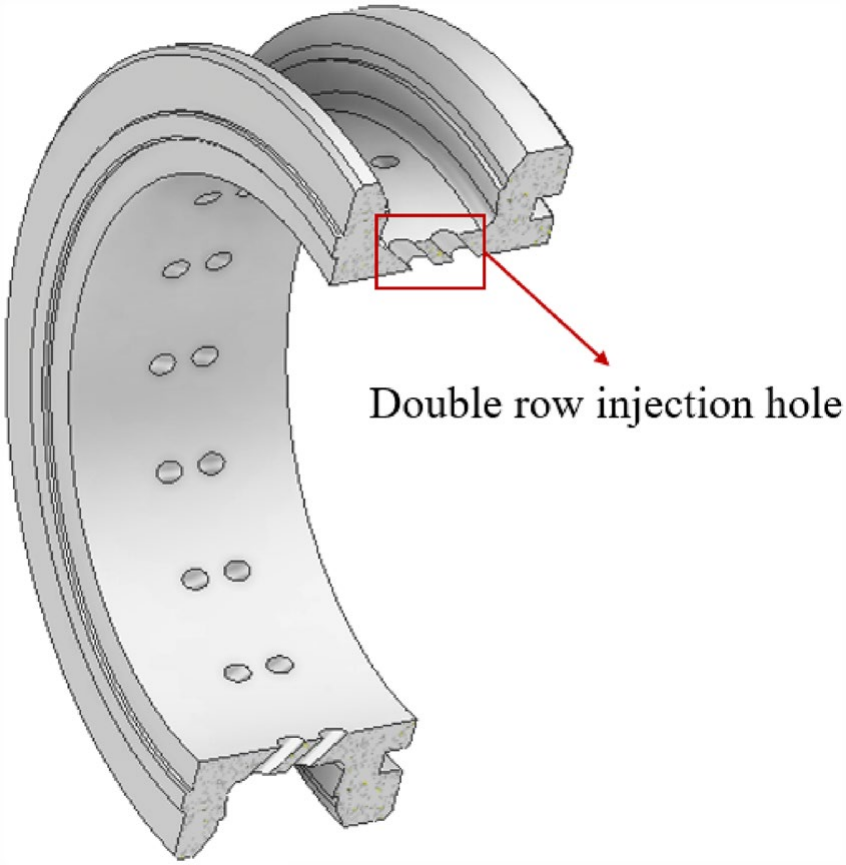
Secondary injection components (axial deflection)-1.

**Figure 24:**
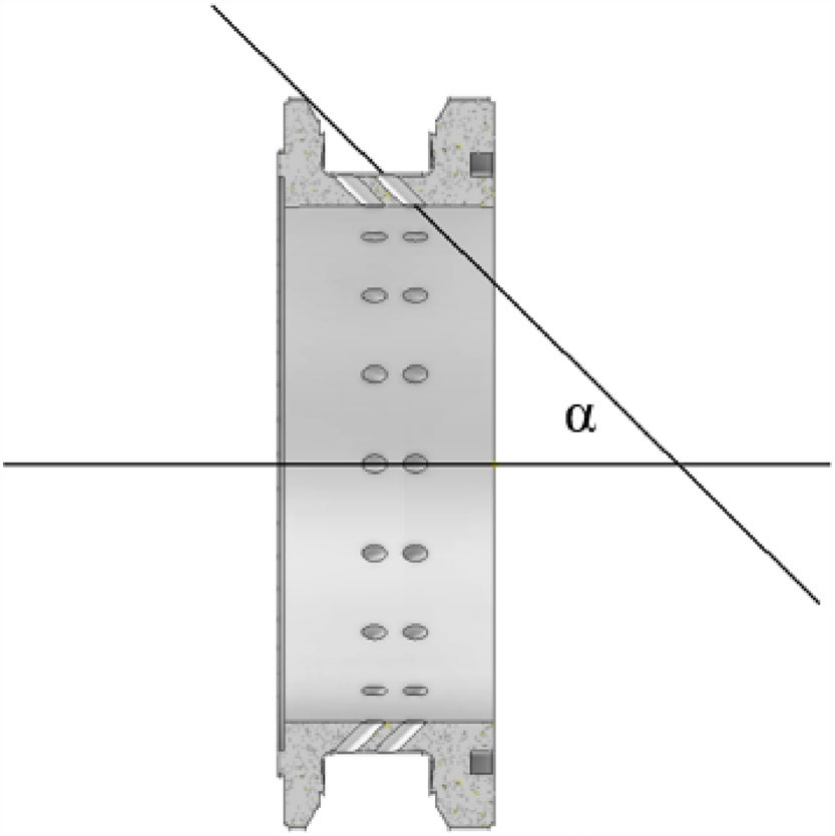
Secondary injection components (axial deflection)-2.

As shown in Figs. 23 and 24, the central injection component (axial deflection). The central injection component (axial deflection) has two rows and 36 injection holes, as shown in Fig. 23. The angle a represents the angle between the injection hole and the axial direction, and four injection components with a values of 0°, 15°, 30°, and 45° were designed. According to Table 9, it is the simulation condition of the simulation for the secondary injection for axial deflection.

**Table 9:** Simulation condition.

Number	Proportion of secondary injection flow (%)	Injection angle (°)
1	65	15
2	65	30
3	65	45
4	65	0

##### Pre-combustion chamber cross-sectional temperature cloud

**Figure 25:**
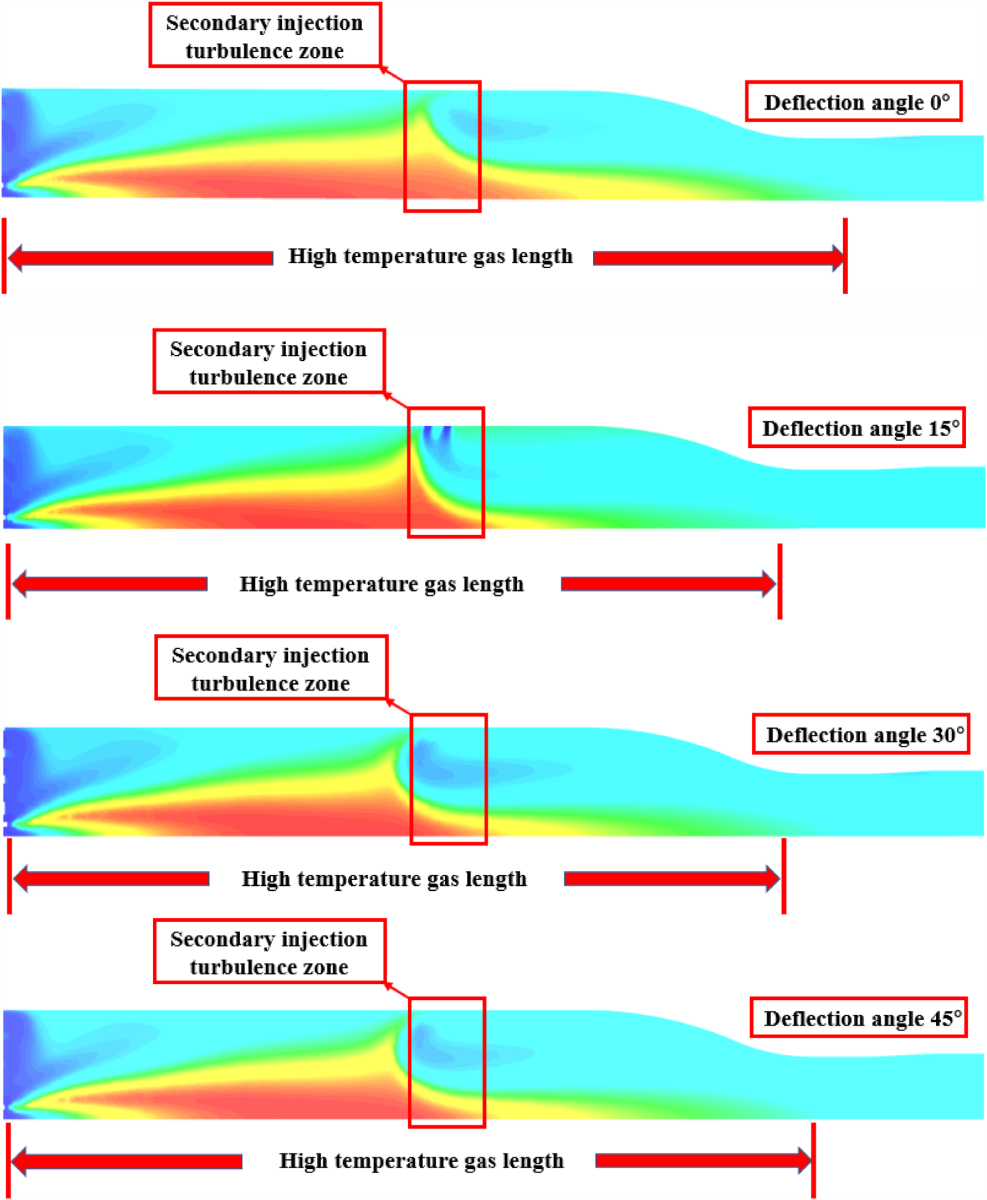
Pre-combustion chamber internal flow field temperature cloud diagram.

As Fig. 25 shown, when the injection angle of 15° and 30°, the secondary injection of high-temperature incoming flow obstruction effect is obvious, and when the secondary injection deflection angle of 45°, it can be seen that the secondary injection of obstruction effect becomes worse, a large number of high-temperature gas flow through the secondary injection section, making the outlet gas temperature uniformity becomes poor.

##### Pre-combustion chamber outlet temperature cross-sectional cloud diagram

**Figure 26:**
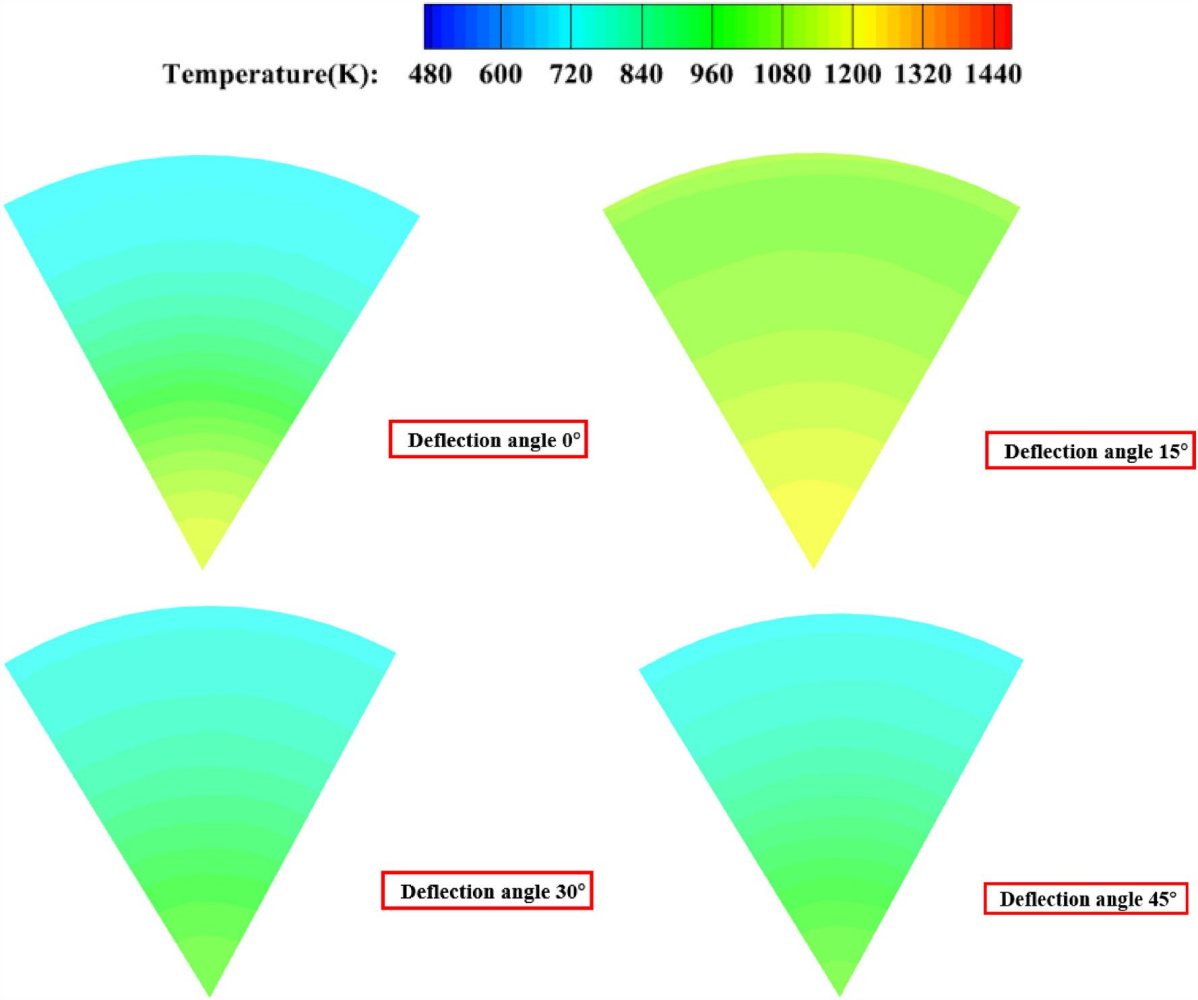
Pre-combustion chamber outlet temperature cross-sectional cloud diagram.

As Fig. 26 shown, when the secondary injection deflection angle is 15° and 30°, the temperature uniformity of the pre-combustion chamber outlet gas is better, while when the secondary injection deflection angle is 45°, the temperature uniformity of the pre-combustion chamber outlet gas is poor, and the middle yellow part can reach 1200 K, and part of the high temperature gas stream is ejected from the pre-combustion chamber outlet.

##### Pre-combustion chamber internal flow field distribution cloud diagram

**Figure 27:**
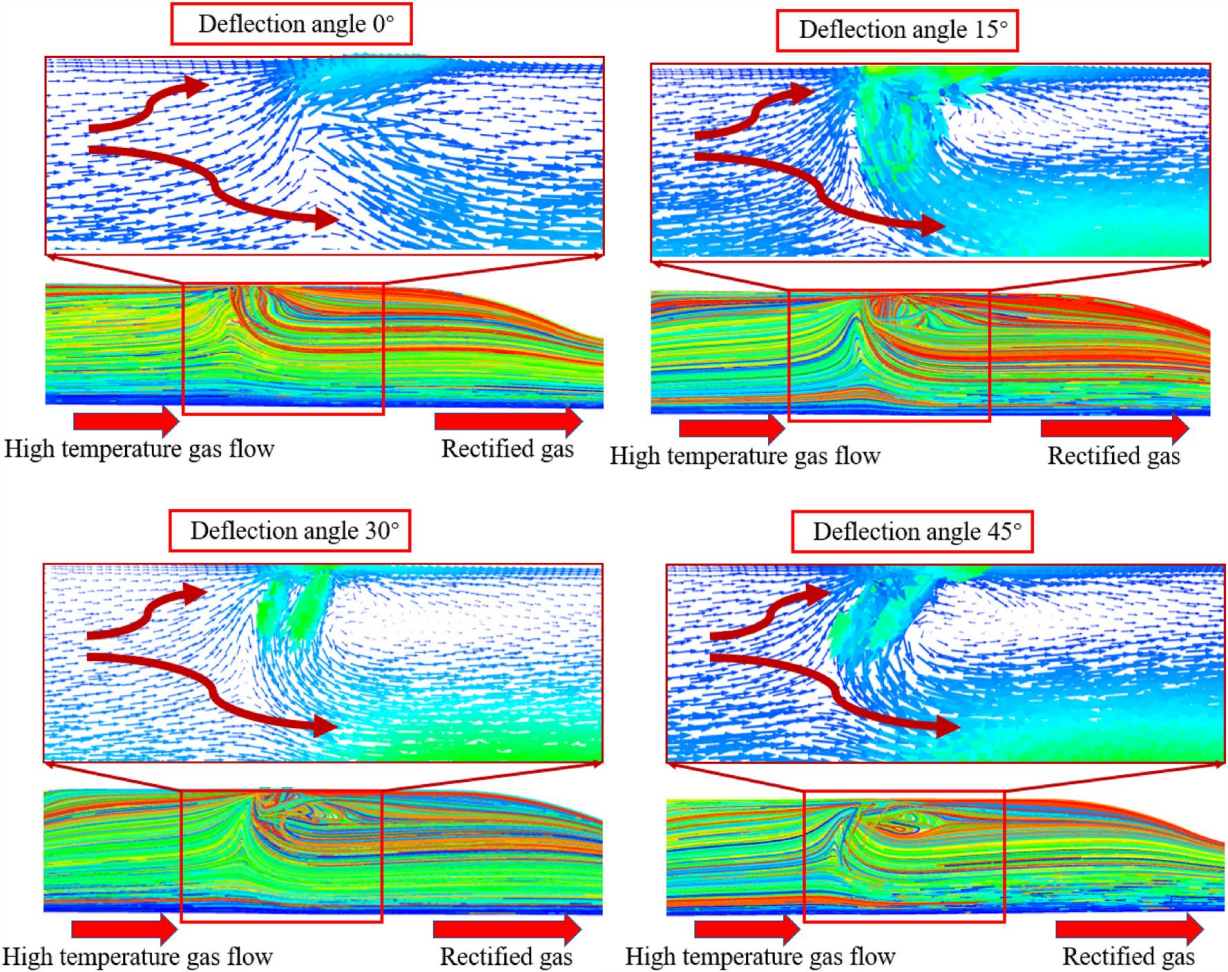
Pre-combustion chamber internal flow field distribution cloud diagram.

**Figure 28:**
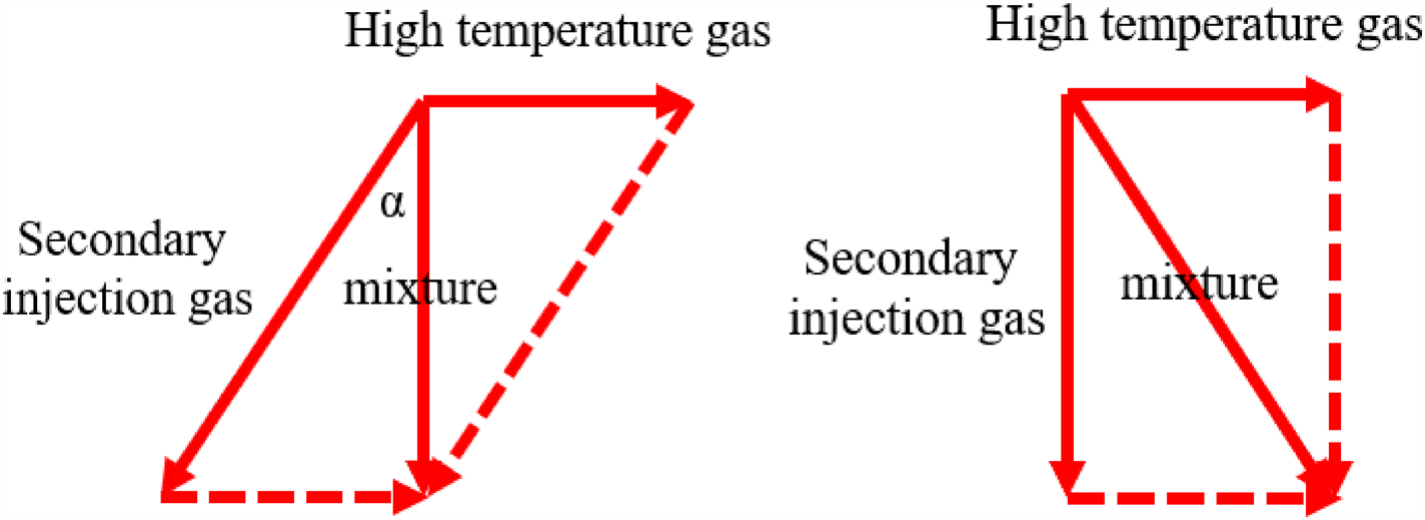
Schematic diagram of velocity vector sum.

As shown in Fig. 27, as the secondary injection angle increases along the axial deflection angle, there is no significant change in the internal flow field of the engine. A portion of the high-temperature gas flows towards the wall and follows the secondary injection gas, while a portion is squeezed to the center of the engine and ejected. However, it can be observed that as the angle increases, the angle of the mixture of the secondary injection gas and high-temperature gas changes from facing the engine outlet to facing the engine head. When the deflection angle is 30°, the angle of the mixture is exactly perpendicular to the axis direction. It can be considered that the radial component of the mixture velocity is zero at this time, making it more effective in blocking high-temperature gas and achieving the best uniformity of outlet gas temperature. The schematic diagram of the velocity vector is shown in Fig. 28.

By selecting the temperature distribution points on one radius of the pre-combustion chamber outlet section, the following temperature distribution profile was plotted.

**Figure 29:**
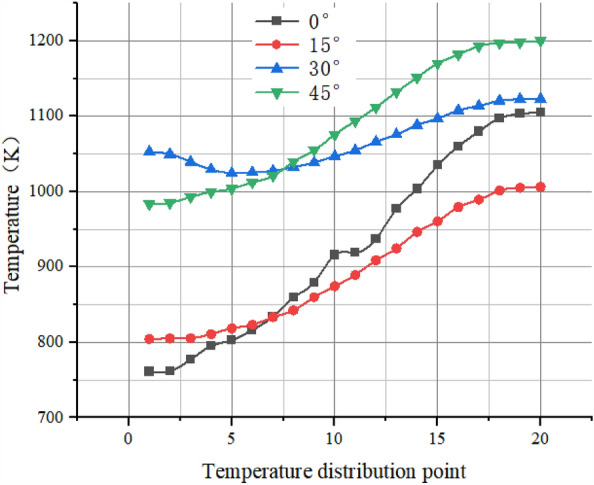
Axial deflection temperature distribution diagram of secondary injection.

As Fig. 29 shown, compared with the deflection angle of 0°, the circumferential deflection of the injection angle will be more favorable to the pre-combustion chamber gas uniformity, where the curve is smoother at a deflection angle of 30°. Among them, the gas temperature uniformity is better.

As shown in Table 10, when the secondary injection deflection angle of 15°, 30° and 45°, the outlet temperature distribution coefficient and standard deviation are less than the deflection angle of 0°, when the secondary injection deflection angle of 30°, the minimum temperature distribution coefficient of the pre-combustion chamber outlet temperature of 0.0467, the standard deviation of 36, the secondary injection deflection angle of 30° will have an excellent optimization effect on the temperature uniformity of the pre-combustion chamber outlet And for the secondary injection deflection angle of 15° and 45°, the optimization effect is not as good as 30°.

**Table 10:** Simulation result data.

Serial number	Secondary injection Deflection angle (°)	Static temperature (K)	Outlet temperature Distribution coefficient	Standard deviation
1	15	894	0.1298	76
2	30	1070	0.0467	36
3	45	1090	0.1009	82
4	0	926	0.1879	121

## Conclusion

The following main findings and conclusions were obtained from the study.

1. Secondary injection will create a turbulent zone in the center of the engine, where high-temperature airflow and secondary injection gas exchange heat and hinder the axial movement of high-temperature gas in the turbulent zone, reducing the axial length of high-temperature gas and optimizing the uniformity of outlet gas temperature.
2. This article found that when the secondary injection angle is deflected in a circumferential direction, as the deflection angle increases, the swirl gradually increases, which will gradually cause the engine gas to rotate and optimize the gas temperature uniformity at the same radius. However, due to the large axial velocity, the secondary injection has little resistance to high-temperature gas and weakens the optimization effect on the gas temperature uniformity in the radial direction.
3. This article found that when the axis of the secondary injection angle is deflected, the gas uniformity is best when the combined velocity of the secondary injection gas velocity vector and the incoming high-temperature gas velocity vector is perpendicular to the axis direction.
4. For the engine in this article Pre-cooled engine pre-combustion chamber of the best combustion organization scheme to choose the secondary injection axial injection angle of 30°, the circumferential injection angle of 0°, the secondary injection flow rate of 65% of the pre-combustion chamber gas blending most complete, the outlet gas temperature uniformity is the best.
5. Based on the experimental results, it can be concluded that the secondary injection structure is limited by factors such as propellant type, flow rate, and test system load, which weakens the effect of secondary injection on the gas uniformity of the combustion engine.

## Data Availability

All data included in this study are available upon request by contacting the corresponding author.
